# Supplementary material for: How do befriending interventions alleviate loneliness and social isolation among older people? A realist evaluation study
Source: PLoS One. 2021 Sep 9;16(9):e0256900. doi: 10.1371/journal.pone.0256900 (PMC8428774; doi:10.1371/journal.pone.0256900)
Supplement: S1 File — (DOCX) [file pone.0256900.s001.docx]

**S1 Appendix.** Initial programme theories - Conceptual framework of characteristics of befriending interventions

| **Conceptual framework component** | **IPT** | **IPT explained** |
| --- | --- | --- |
| Matching befrienders and service users | 1a | If the matching process of befrienders and service users takes into consideration their demographics, interests, personality, background, life histories, values and/or experiences (C), then it is likely that a friendship would be established (O) as the high compatibility will trigger a common bond which can facilitate intimacy, reciprocity and mutual trust (M – reasoning). Moreover, the experiential similarity facilitates mutual understanding (M – resource) which will increase the befriender’s ability to deliver a more attuned response to the needs of the service user (O), and will lead to the service user feeling more comfortable in expressing their difficulties with their befriender (M – reasoning). |
|  | 1b  (Rival) | If the befriender has good personal qualities, takes genuine interest in the life of their service user, has good social competence and is also very adaptable (M – resource), then a good quality relationship is likely to be developed between both the parties (O) regardless of the extent of commonalities (C). |
| Face-to-face in-person contact | 2a | If the service user receives face-to-face contact by the befriender rather than via the telephone, then the likelihood of a friendship being developed is strengthened because both parties are better able to gain knowledge of each other in their context with the aid of non-verbal cues (M – resource), and make more accurate judgements (M - reasoning). This can stimulate conversation-making, and reduce the sense of social distance for the service user (M – reasoning) and consequently feelings of loneliness will be reduced (outcome). |
|  | 2b  (Rival) | If the service user receives telephone befriending then they may feel relieved of the stress of having to host the befriender in their home (M - reasoning), particularly for those service users with social anxiety or other psychological problems (C). Moreover, the service user is able to maintain their anonymity, making them feel more comfortable in disclosing private/sensitive information without fear of being judged (M - reasoning). Hence, a meaningful relationship is likely to be developed which will alleviate feelings of loneliness and social isolation in the service user (O). |
| Training of befrienders | 3 | If the befriending service adequately trains befrienders who might or might not have prior relevant experience (C), then the befrienders are likely to become more competent and confident in their role (O). This is because the training provides opportunities for the befriender to develop knowledge and understanding of the target population of the service, and allows them to realise their role and boundaries of the service as well as the resources available to them (M – reasoning). Thus, a good connection is likely to be developed with their service user which will aid in the alleviation of loneliness and social isolation (O). |
| Paid vs unpaid befriending role | 4a | If the service user receives a volunteer befriender (as opposed to a befriender that receives monetary compensation) particularly those who have limited mobility or are housebound (C), then reciprocity in the relationship will be enhanced because the service user recognises and values that their befriender is choosing to spend time with them rather than being under any professional obligation or for financial gain (M – reasoning). The service user feels more comfortable and the engagement is likely to feel more natural and friendship-like which will lead to alleviation in feelings of loneliness (O). |
|  | 4b  (Rival) | If the service user receives a volunteer befriender (as opposed to a befriender that receives monetary compensation), they are less likely to take action should they feel dissatisfied with the service or that their befriender is not the right match for them (O). This is because the service user may feel guilty as they are aware that their befriender is choosing to spend time with them rather than being under any professional obligation to do so (M – reasoning). Consequently, a meaningful relationship is unlikely to be developed between both parties and this increases the likelihood of feelings of loneliness being exacerbated in the service user (O). |
|  | 5a | If the befriender is delivering the service with monetary compensation (C), then there could be an uptake of the befriending role by people who prioritise the monetary benefit more than developing a deep connection with their service user and establishing a meaningful relationship (O). This could lead to a lack of an emotional connection between both parties (O) and the service user feeling undervalued (M – reasoning). Thus, the befriending relationship is likely to remain on a superficial level, potentially leading to an exacerbation in feelings of loneliness (O). |
|  | 5b  (Rival) | If befrienders receive monetary compensation for their role (C), this is unlikely to affect their ability to develop an emotional connection with their service user because the befriender acquires a caring disposition (C) and genuinely wants to positively impact the life of their service user (M – resource). Moreover, the befriending visits and activities would be better tailored to the preferences and interests of the service user (M – resource) and they would likely feel more secure in the befriending relationship (M - reasoning). Hence, the service user would feel more cared for and a trusting and meaningful relationship would be established between both parties, thus reducing feelings of loneliness (O). |
| Duration of service (limited and indefinite length, frequency and time per visit) | 6 | If the befriending service is delivered long-term, especially to service users who are housebound and/or have little or no family/support network and therefore are socially isolated (C), then the regular companionship and social contact provided by the befriender (M – resource) stimulates the development of a meaningful relationship between both parties (O). The befriender and service user are able to develop good knowledge of each without time pressure (O) and the relationship is more likely to feel natural and genuine (M – reasoning). This will lead to a trusting and mutually beneficial relationship being established which will alleviate feelings of loneliness and social isolation in the service user (O). |
| One-to-one vs group delivery method | 7 | If the befriending service is delivered on a one-to-one basis, then the likelihood of a meaningful relationship being established is enhanced because the private interaction eliminates external distractions and provides intimacy and safe environment (M – resource). This facilitates greater stimulation in individualised conversations as the service user is likely to feel more comfortable in disclosing personal matters which enhances the development of trust in their befriender (M – reasoning). This is particularly beneficial in circumstances where the service user has a cognitive impairment and/or lacks social skills needed to thrive in group settings (C). Thus, a meaningful relationship is likely to be developed which will alleviate feelings of loneliness and social isolation in the service user (O). |
| Continuity in the same befriender visiting | 8 | If the service user is visited by the same befriender over time (as opposed to different befrienders), then a reciprocal friendship is likely to be established between both parties because the continuity in the befriending relationship allows for a deeper knowledge to be developed of each other and for a meaningful rapport to be established which will alleviate feelings of loneliness and social isolation (O). Having the same befriender is particularly valuable in circumstances where the service user has a cognitive impairment such as dementia (C) as they are more likely to feel a sense of security (M – reasoning) and mutual trust would be developed over time as familiarity strengthens (O). |

**S2 Appendix.** Characteristics of befriending service cases

| **Case** | **Service aim** | **Location** | **Target population** | **Type of befriending relationship and delivery method** |
| --- | --- | --- | --- | --- |
| A | To help people living with dementia to continue to play active roles in their communities, to help them to lead fulfilling lives, and reduce feelings of loneliness and social isolation. | NI wide and in other parts of the UK | People with dementia | Open-ended with a volunteer befriender. Delivered in-person on a one-to-one basis. |
| B | To reduce social isolation and/or loneliness experienced by older people living in their own homes, have little contact with family and friends, and are unable to attend day centres or other social groups due to older age and age-related illnesses. | Belfast | General population of older people aged 60 years and above |  |
| C | To provide a person-centred service and support individuals to access social opportunities in the community in order to reduce feelings of loneliness and social isolation. | Belfast | People with learning, physical or sensory disability | Length of befriending relationship is dependent on the hours of befriending support purchased by service users.  Delivered in-person on a one-to-one basis. |
| D | To help older people who have become isolated as a result of their health condition and to get them back into community life. | Across NI and in Narin (Scotland) | Older adults aged 60 and above with a long-term health condition | Fixed term befriending relationship of eight weeks with a volunteer befriender. Delivered in-person on a one-to-one basis. |
| E | To improve the quality of life for individuals with sight and/or hearing loss, by reducing feelings of loneliness and isolation they endure through befriending. | NI wide and in other parts of the UK | People with sight and/or hearing loss | Open-ended with a volunteer befriender. Delivered in-person, via telephone and email on a one-to-one basis. |

****S3 Appendix.** Topic guide for service personnel**

| **No.** | **Question** | **Rationale** |
| --- | --- | --- |
|  | I understand that you are a manager of a befriending service, can you tell me how long you have been in this role for? | Questions 1 and 2 are introductory. |
|  | What was your previous role and what did it entail? |  |
|  | In what ways do you think the befriending service has been of benefit to the service users? | Looking for known, unknown intended and unintended outcomes. |
|  | Do you think that the outcomes have been the same for all service users, including those with mental or physical health conditions? Why?  If no, in what ways have they been different? | This question seeks to identify ‘in what respects, for whom’ and ‘to what extent, for whom’ the outcomes (known, unknown intended and unintended) are achieved. |
|  | You mentioned that the befriending service has benefited the service users by XXXXXXX, do you think this made them to feel less lonely and isolated? Or did it have other outcomes? | Looking for known, unknown intended and unintended outcomes. |
|  | Is the goal of the service to provide friendship or is it to improve confidence and promote independence so that the service user can link back to their community? | This question aimed to identify the goal of the befriending service. |
|  | Is there a given period when the service user will begin to withdraw from the service? | Questions 7 and 8 aimed to identify if the befriending service had a limit on the number of sessions a service user is entitled to and if there were any policies regarding withdrawing from the service. |
|  | Is there a policy about when someone withdraws from the service? |  |
|  | In what ways do you think the befriending service has been of benefit to the befrienders? | Looking for known, unknown intended and unintended outcomes of the befriending service for the befrienders. |
|  | Were both the befriender and service user clear of the boundaries? | Exploring potential contexts. |
|  | Could you explain to me the types of people and places where this service may be more effective? | Questions 11 and 12 are exploratory questions to identify the ‘for whom and in what circumstances’ befriending services work better. It aimed to identify and explore different contexts. |
|  | Who would the service not work for? Why? |  |
|  | How do you think the befriending service works for the service user? | Looking for mechanisms (i.e. how service personnel think service users respond to the befriending service. |
|  | Do you think it is important for the befrienders to be matched to the service users? Why? | Looking for mechanisms about why matching works (i.e. how do service users and befrienders respond when a good match is produced). Also aimed to identify outcomes (intended and unintended). |
|  | Why is it important to match a befriender to the service user? |  |
|  | How are the befrienders and the service users matched?  Prompts: What are the key features for matching the befriender to the service user? | Questions 16 and 17 aim to identify if service personnel use a specific template to guide the matching process. |
|  | Do you use a specific template to match the befrienders to the service users? |  |
|  | Does matching always take place or have there been cases where it was not possible to match a befriender to a service user? Why was this? | Looking for intended and unintended outcomes. |
|  | What is the criteria for selecting befrienders? What characteristics do you look for? | Questions 19 and 20 are exploring contexts (characteristics of the befrienders). |
|  | What skills and qualities do the befrienders need to have? |  |
|  | One of the ideas we have is that befriending provides personal, social and emotional support, do you think that this is important to reduce loneliness? Why? | Questions 21 and 22 are looking for mechanisms (i.e. how the support provided by the befriender affects how service users respond to the service (i.e. reasoning, feeling, behaviour and attitude). |
|  | Do you think that it is easier to provide social and emotional support when there is a match? Why? |  |
|  | What do you think are the benefits of delivering the befriending service in-person? Do you think it makes a difference? | Looking for mechanisms (i.e. how in-person interaction contributes to how the befrienders and service users respond to the service (i.e. reasoning, feeling, behaviour and attitudes). |
|  | Do you think that telephone befriending would be more effective? Why? | This question was asked with aims to identify information that could be compared with data gathered from Q23 regarding in-person delivery, in order to identify the effectiveness of both methods. |
|  | Befriending services often provide one-to-one contact, do you think this method of service delivery is important? Why? | Looking for mechanisms (i.e. how receiving one-to-one contact with the befriender affects how the service user responds to the service (reasoning, feeling, behaviour and attitude)). |
|  | Do you think that the service users will benefit from group contact? | This question aimed to identify information that could be compared with data gathered from Q25. |
|  | If the befrienders of the service are volunteers, ask: ‘I’m aware that the befrienders are volunteers, do you think that it makes a difference that they are volunteers? How?’ | Questions 27 and 28 aimed to identify if the befrienders of the service are paid staff or volunteers. Also, it explores mechanisms (i.e. how service personnel think that service users respond (feel, reason, behave) to befrienders if they are paid/volunteers). |
|  | If the befrienders of the service are paid workers, ask: ‘Do you think it makes a difference that they are paid? If so, how?’ |  |
|  | Does it matter if the same befriender visits the service user? | Looking for mechanisms (i.e. how do service personnel think that service users respond to the same befriending visiting them). |
|  | What do you think makes a successful befriending relationship between the befriender and the service user? | This question aimed to ascertain the key components service personnel believe are necessary to the success of a befriending relationship between the befriender and the service user. |
|  | What do you think makes a successful befriending service? | This question aimed to ascertain the key components service personnel believe are necessary to the success of a befriending service. |
|  | What is it about the way [INSERT NAME OF BEFRIENDING SERVICE HERE] works that makes a difference to how it works? | This question aimed to understand how the implementation of the befriending service affected the mechanisms produced and therefore outcomes. |
|  | We’ve seen that befriending service works differently in different places. What is it about this place that makes it work so well or less well? | This question aimed to elicit understanding about how the context affects the outcomes by affecting which mechanisms fire. |
|  | If you could change something about this befriending service to make it work more effectively, what would you change and why? | This question aimed to elicit understanding of why the befriending service has worked or not worked as effectively, as well as identifying strategies for improvement. |
|  | What else do you think we need to know, to really understand how this befriending service has worked here? | This question probes participants to comment on anything that was not covered in the interview. The structure of the question keeps the focus on ‘how the befriending service works’ and ‘in this context’. |

****S4 Appendix.** Topic guide for befrienders**

| **No.** | **Question** | **Rationale** |
| --- | --- | --- |
|  | I understand that you are a befriender, can you tell me how long you have been befriending for? | This question is introductory. |
|  | Can you tell me why you became a befriender? | The purpose of asking this question was to identify potential contexts and mechanism. For example, if the target population of the befriending service was individuals with a health condition, this question would identify if the befriender joined the role because they had a personal experience of caring for someone with the same health condition. From this, the researcher explored possible mechanisms of how this experience impacted their role as a befriender. |
|  | If they have personal experience of caring for someone with a condition that the service offers befriending for, ask them:  In what ways do you think that you having experience of caring for someone with [insert condition here, e.g. dementia] impacted your role and ability as a befriender? | This question is a follow-up of question 2 and was only applied if the befriender mentioned that they had taken the role based on their personal experience with aims to make a positive difference to someone with the same health condition that their relative or friend has/had. The question is looking for mechanisms (i.e. how has their caring experience (resource) influenced their role as a befriender (response)). |
|  | In what ways do you think the befriending service has been of benefit to your service user?  Prompts: Are there any other benefits to the person receiving the service, e.g. confidence, self-esteem, quality of life? Give some examples. | Looking for known, unknown intended and intended outcomes of the befriending service. |
|  | How do you think that providing this service to them give them more ‘[state benefit here]? E.g. what is it about you accompanying them to outdoor activities that makes them more confident? Why would they become more confident as a result of this? | This question is a follow up of question 4 and aims to explore the mechanisms (i.e. how befrienders think that providing companionship to their service user (resource) affects the reasoning of the service user (response)). |
|  | Do you think that the outcomes have been the same for all service users, including those with mental or physical health conditions?  If yes, why?  If no, in what ways have they been different? | This question seeks to identify ‘in what respects, for whom’ and ‘to what extent, for whom’ befriending services achieve their outcomes. |
|  | In what ways did you help your client to link to the community? | This question aimed to identify if the goal of the befriending relationship is to link the client back to the community or if it is more to provide companionship to the service user. |
|  | Have you been matched to a service user? | This question aimed to identify if matching was carried out in the befriending relationship. |
|  | How were you matched to the service user? What was the criteria? | This question is a follow-up of question 8 and aimed to identify how the matching process was conducted. |
|  | Do you feel like you connect well with your service user? Why? | Looking for mechanisms, and intended and unintended outcomes of the befriending service. |
|  | Do you consider yourself to be a friend to your service user? Is your service user your friend? Why? | Added as a result of existing literature comparing befriending relationships to friendships. This question aimed to obtain the perspectives of befrienders to identify if they considered their service users to be their friend. Thus, the question is looking for mechanisms, and intended and unintended outcomes of the befriending relationship. |
|  | You mentioned that the befriending service has helped your service user by [insert benefit mentioned previously], do you think this made them to feel less lonely and isolated or did it have another outcome? | This question aims to identify the intended and unintended outcomes of the befriending service. |
|  | How have they demonstrated this? | This question is a follow up of question 12 and is exploring the mechanisms of the befriending service (i.e. how has the reasoning, behaviour, attitudes and beliefs of the service user changed as a result of receiving the befriending service). |
|  | Could you explain to me the types of people and places where this service may be more effective? | Exploratory question to ascertain for whom and in what circumstances befriending services work by exploring the characteristics (contexts) of the service users. |
|  | What do you think are the benefits of delivering the befriending service in-person? Do you think it makes a difference? | Looking for mechanisms (i.e. how in-person interaction benefits both befriender and service user (reasoning)). |
|  | Have you ever delivered telephone befriending? | Questions 16 and 17 aim to obtain information needed to compare befriending delivered both telephone and in-person in terms of the effectiveness of the contact method. |
|  | Would you rather deliver the befriending via telephone? Why? |  |
|  | Does delivering the befriending service have any impact on you? If so, what?  Prompts –  -Changed your outlook on life  -Changed your view of older people | This question aimed to identify the intended and unintended outcomes of befriending service on the befriender (i.e. if the befriender has become a more positive person as a result of their role). |
|  | Do you find being a befriender rewarding? Why? | Looking for mechanisms (i.e. befriender’s reasoning of their role). |
|  | Did you undergo training before becoming a befriender? | Questions 20-23 aim to identify if training is a component of the befriending service and how the training was delivered. Moreover, it aimed to identify mechanisms (i.e. how training affected the befriender’s reasoning, attitude and behaviour). |
|  | Were they online or in-person? Which would you prefer? |  |
|  | Do you receive regular up-to-date training? |  |
|  | Do you feel that the training you received to be a befriender was adequate? Do you feel that you needed more training? If so, on what? |  |
|  | Did you have a clear understanding of your role as a befriender and of what the boundaries of your role were? | Exploring potential contexts. |
|  | Do you feel as though you receive enough support as a befriender? Why? | Looking for known intended and unintended outcomes. |
|  | How many people do you provide the befriending service to at any one time? | This question aimed to identify if the befriender is delivering the service to more than one service user. |
|  | How often do you see your service user (in hours per week/no. of visits etc.)? | This question aimed to identify the duration (length and frequency) of the befriending visits. |
|  | Do you feel this is enough? Do you provide any additional input beyond the time you are supposed to? |  |
|  | What activities do you undertake with your service user? Do they suggest the activities or do you decide for them? | Looking for potential outcomes. |
|  | What do you think makes a successful befriending relationship? | This question aimed to identify the befriender’s reasoning of the key components that contribute to successful befriending relationship. |
|  | One of the ideas we have is that befriending provides personal, social and emotional support, do you think that this is important to reduce loneliness? Why? | Questions 31 and 32 are looking for mechanisms (i.e. how befrienders think that their service user respond to the support provided by the befriender). |
|  | Do you think that it is easier to provide social and emotional support because you are matched? Why? |  |
|  | Do you think it makes a difference that the service user always sees the same befriender? Why? | Looking for mechanisms (i.e. how do befrienders think that service users respond to the same befriender visiting them). |
|  | Befriending services often provide one-to-one contact, do you think this method of service delivery is important? Why? | Looking for mechanisms (i.e. how befrienders think that service users respond to the one-to-one delivery method). |
|  | What would you say would be the benefits of group delivery? | This question aimed to identify information that could be compared with data gathered from question 34. |
|  | Do you get paid to be a befriender?  If No, ask: Some befrienders are paid, why do you engage when there isn’t payment available?  Would you rather be paid for this service? Why?  If Yes: Do you think that you’d still be interested in providing befriending if that payment wasn’t available? Why? | This question aimed to identify if the befriender is a paid staff or a volunteer. Also, the question is exploring mechanisms (i.e. how are befrienders responding to being a volunteer/paid staff). |
|  | If you could change something about this befriending service to make it work more effectively here, what would you change and why? | This question aimed to elicit understanding of why the befriending service has worked or not worked as effectively, as well as identifying strategies for improvement. |
|  | What else do you think we need to know, to really understand how this befriending service has worked here? | This question probes participants to comment on anything that was not covered in the interview. The structure of the question keeps the focus on ‘how the befriending service works’ and ‘in this context’. |

****S5 Appendix.** Topic guide for service users**

| **No.** | **Question** | **Rationale** |
| --- | --- | --- |
|  | I understand that you use the befriending service, can you tell me how long you have been using the service for? | This question aimed to identify if the befriending service has a maximum number of sessions a service user can receive, i.e. maximum of eight sessions. |
|  | Why did you start to use this service? | This question aimed to explore potential contexts (i.e. loss of a loved one, lack of family support or social network, health condition/diagnosis). From this, the researcher explored possible mechanisms of how this experience impacted them as a service user of the befriending service. |
|  | Was the service what you expected? Has it met your expectations? | This question aimed to identify potential outcomes of the befriending service, whether positive or negative. |
|  | Were you aware of the boundaries of the service before you started receiving it? | Exploring potential contexts. |
|  | Sometimes befriending services are used for people who feel quite lonely or isolated. Do you think that you felt lonely or isolated? | Exploring potential contexts (loneliness and social isolation). |
|  | Since using the service, do you still feel lonely or isolated or did it have a different outcome? | Looking to identify outcomes of the befriending service and the nature and extent of the outcome. |
|  | How has the befriending service helped you?  For example, do you feel confident enough to socialise in your local community as a result of having a befriender? Why? | Looking for mechanisms, and the intended and unintended outcomes of the befriending service. |
|  | Do you feel like you are part of your community? Why? | Looking for known intended outcomes of the befriending service. |
|  | Do you feel like you connect well with your befriender? Why? | Looking for mechanisms to explain why the befriending relationship is successful. |
|  | Would you consider your befriender to be a friend? Why? | This question is looking for mechanisms and intended and unintended outcomes. It was added as it was identified through literature that some befriending services have a goal to provide companionship to service users and not necessarily friendship. However, friendship between the service user and the befriender can form as a result of the similarities or complementing differences between the befriender and the service user. |
|  | You told me that the service is valuable for you because it helped you by XXXXXXXXX, do you think that everyone using this service feels the same? Or do you think that other people might benefit in different ways? | Looking for potential contexts (i.e. characteristics of service users). This question aimed to identify specific information about ‘for whom’ the befriending service might or might not be effective for. |
|  | There are lots of ideas about how befriending services actually work, and we think that they work differently in different places or for different people. Who do you think the service works for and why? | The purpose of this question is to identify potential contexts. It aimed to identify if the service works differently for different people. |
|  | In what ways might other people benefit from this befriending service? | The aim of this question is to identify the intended and unintended outcomes of the befriending service. |
|  | Have you ever received telephone befriending?  If yes, Ask: How did you feel about this?  If no, Ask: Would you have liked to receive telephone befriending? Why? | The aim of this question is to identify information on telephone befriending that can be compared with befriending delivered in-person to aid in identifying mechanisms of in-person befriending. |
|  | How important is it that the service is delivered in-person? Why is this important? | Looking for mechanisms (i.e. how do service users respond to the in-person interaction of the befriending service). |
|  | Is it the same befriender that visits you? Do you prefer this or would you rather have different befrienders visiting you? Why? | Looking for mechanisms (i.e. how do service users respond to having the same befriender visiting them). |
|  | Do you know if your befriender is paid or if they are a volunteer? | The aim of this question is to identify if the service user is aware if their befriender is a volunteer or a paid staff. |
|  | Would you prefer if your befriender was paid? Or would you prefer a volunteer? Why? | Looking for mechanisms, and the intended and unintended outcomes of the befriender being either a voluntary worker or a paid staff. |
|  | Do you think that receiving a volunteer befriender/paying for a befriender, has contributed to how the service benefits you? Why? | This question is a follow-up of question 18 and the aim is to get a more in-depth response on why the service user either values their befriender being a volunteer or being a paid worker. |
|  | Do you receive the service for free or do you pay for it?  If service users do not pay, ask: There is no expected changes to the service but if you did have to pay, is it a service that you’d want to pay for? Why?  If service users pay, ask: Would you rather not pay for the service? Why? | This question aims to explore outcomes of the befriending service. |
|  | Do you do any activities with your befriender? If so, are you the one that suggests these activities or does your befriender suggest them? | Looking for mechanisms (i.e. how service users respond to the activities undertaken with their befriender (resource)). |
|  | What type of activities do you do? | This question is a follow-up of question 21. |
|  | Would you like to do more activities with your befriender? If so, what activities? | This question is a follow-up of question 22. |
|  | How often do you see your befriender and for how long do they stay for? Are you happy with this or would you like to see them more often? | The service user is likely to be most knowledgeable about the importance and impact of ‘duration’ of the befriending service, i.e. the length of the service and the frequency of the visits from the befriender. This question aimed to identify mechanisms (i.e. how service users respond to the length and frequency of the befriending service). |
|  | Were you matched to a befriender? | This question aimed to identify if matching was carried out in this befriending relationship. |
|  | Does being matched to a befriender make a difference? Is it important to be matched to a befriender? Why? | Looking for mechanisms (i.e. how service users’ reasoning, attitudes and behaviour, changed as a result of the match with the befriender). |
|  | Do you have a good relationship with your befriender? Why? | This question is looking for unintended and intended outcomes. It aimed to confirm if a successful match between the befriender and the service user is a key ingredient to the success of a befriending relationship. |
|  | Befriending services often provide one-to-one contact, do you think this method of service delivery is important? Why?  Would you prefer if the service was delivered in groups? Why? | Looking for mechanisms (i.e. how service users respond to the one-to-one delivery method of the befriending service). |
|  | Do you like how the befriending service is delivered to you? Why? | Questions 29 and 30 aimed to elicit understanding of why the befriending service worked (or not) as effectively and strategies for improvement. |
|  | Is there anything you would change? |  |
|  | What else do you think we need to know, to really understand how this befriending service has worked for you? | This question probes participants to comment on anything that was not covered in the interview. The structure of the question keeps the focus on ‘how the befriending service works’ and ‘in this context’. |

****S6 Appendix.** Topic guide for family member of service user**

| **No.** | **Question** | **Rationale** |
| --- | --- | --- |
|  | I understand that you have a relative who receives support from a befriending service, can you tell me how long they have been using the service for? | This question is introductory and aims to get the family member talking and to identify how long the service user has been receiving the befriending service. |
|  | Why did they start using the service? | This question aimed to explore potential contexts (i.e. loss of a loved one, lack of family support or social network, health condition/diagnosis). |
|  | How did they get referred on to the service? | This question aimed to identify how service users were enrolled for the befriending service. |
|  | Do you think that the befriending service has had any impact on your relative?  *Prompts*  Has the service helped your relative in any way? How? (e.g. relative is more confident/happy) | Looking for the intended and unintended outcomes of the befriending service. |
|  | In what ways might other people benefit from the befriending service?  *Prompt*  For whom might the befriending service work differently for? Why? | This question aimed to identify ‘in what respects, for whom’ and ‘to what extent, for whom’ the befriending service achieves its outcomes. |
|  | You mentioned that the befriending serviced has helped your relative by [insert stated benefit(s)], do you think this has made them to feel less lonely and isolated or did it have any other outcomes? | This question aimed to identify the intended and unintended outcomes of the befriending service. |
|  | Do you receive a paid befriender or a volunteer? | This question aimed to identify if the family member is aware if the befriender for their relative (service user) is a volunteer or a paid staff. |
|  | What would you say are the benefits of having a paid befriender that might not come with a volunteer befriender? | Looking for mechanisms (i.e. how the relative (service user) reacts (e.g. feels, reasons, beliefs, attitudes and behaviour) to the paid befriender/volunteer). |
|  | Do you think that the in-person delivery method of the befriending service is the best method? Why? | Looking for mechanisms (i.e. how the family member thinks that their relative (service user) responds to the in-person interaction of the befriending visits). |
|  | Do you think that your relative might have benefited from telephone befriending? Why? | The aim of this question is to identify information on telephone befriending that can be compared with befriending interventions delivered in-person (Q9) to aid in identifying mechanisms of in-person befriending. |
|  | How many hours of befriending does your relative receive per week? Do you think that this is sufficient? | This question aimed to identify the duration (length and frequency) of the befriending visits. |
|  | Do you think that it is important for the befriender to have been matched to your relative? If so, why?  *Prompt*  Do you think it is important for the befriender to share something in common with your relative? | Questions 12 and 13 are looking for mechanisms (i.e. how family members believe that their relative (service user) responded (reasoning, attitudes and behaviour) as a result of the match with the befriender). |
|  | Do you think that your relative would have gotten along with the befriender if there was not a match or a good match? Why? |  |
|  | Do you think that having the same befriender visiting your relative is important? Why? | Questions 14 and 15 are looking for mechanisms (i.e. how family members think that the service user responds to having the same befriender visiting them). |
|  | Do you think it would make a difference if different befrienders visited your relative? |  |
|  | We’ve seen that this befriending service works differently in different places. What is it about how it is delivered here that makes it work so well or less well? | The aim of this question is to elicit understanding about how the context affects the outcomes by affecting which mechanisms fire. |
|  | What factors do you think has caused the befriending service to be effective/ineffective?  *Prompts*  -Consistent support  -Motivated befrienders | Questions 17 and 18 aim to elicit understanding of why the befriending service has worked or not worked as effectively, as well as identifying strategies for improvement. |
|  | If you could change something about this befriending scheme to make it work more effectively for your relative, what would you change and why? |  |
|  | What else do you think we need to know, to really understand how this befriending service has worked for your relative? | This question probes participants to comment on anything that was not covered in the interview. The structure of the question keeps the focus on ‘how the befriending service works’ and ‘in this context’. |

**S7 Appendix.** Refined programme theories across cases

| **PT 1:** The matching process between a befriender and service user needs to consider their respective socio-demographic characteristics (e.g. age, gender, locality, health status), and their interests, beliefs, and experiences (mechanism – resource), particularly in circumstances where service users have a sensory, cognitive or physical impairment (context). Matching these characteristics will increase the likelihood of stimulating the development of trust (mechanism – reasoning), and reinforcing the dispositions of befriender and service user (mechanism – resource), thereby facilitating reciprocity, and providing opportunities for a service user to engage in health and wellbeing-enhancing activities. Moreover, shared experiences between befriender and service user (related to, for example, health or bereavement) increases: understanding about the challenges and difficulties that a service user faces (mechanism – resource); their capacity for empathy (mechanism – reasoning); and the delivery of a response that is more attuned to the service user’s needs (outcome). Service users are more likely to feel understood, more comfortable about expressing their emotions, and confiding in their befriender (mechanism – reasoning). Thus, a meaningful relationship between service user and befriender is likely to develop and evolve into a type of friendship, which will alleviate feelings of loneliness in a service user (outcome). However, if a befriender has good personal qualities (e.g. kind personality), takes genuine interest in the life of their service user, has good social competence (e.g. good communication skills) and is also very adaptable (mechanism – resource), then a good quality relationship is likely to be developed between both parties (outcome) regardless of any lack of commonalities in characteristics or experiences (context). The differences in characteristics can act as a conversation stimulant (mechanism – resource). |
| --- |
| **PT 2:** If person-to-person communication is provided to service users, particularly those with a sensory or cognitive impairment which can affect their social and communication skills (context); then a befriender is able to observe non-verbal cues such as body language, eye-contact and empathy (mechanism – resource), and therefore adapt the delivery of the service accordingly with use of visual aids where necessary (e.g. sign language) (mechanism – resource) to assist in effective communication (outcome). Moreover, the in-person interaction allows befriender to utilise non-verbal communication techniques such as touch (mechanism – resource), assist with small practical tasks and also engage service users in games and activities of interest inside/outside of their home (where feasible). This can provide cognitive stimulation (mechanism – resource), facilitate the feeling of belonging in their community (mechanism – reasoning) and create opportunities for service users to expand their social network (mechanism – resource). Additionally, service users are likely to feel more secure with their befriender and reassured that they are the main focus of engagement (mechanism – reasoning) as they can see that their befriender is not distracted (mechanism – resource). Reciprocity is also likely to be established (outcome) as befriender can see how they are impacting the life of service users which is rewarding and can motivate them to continue delivering the service (mechanism – reasoning). However, if a service user receives telephone befriending, then they are relieved of the stress of having to host a befriender in their home (mechanism – reasoning) because of hoarding, social anxiety or other psychological problems (context). Moreover, the lack of external factors as well as the confidentiality promoted via this communication method (mechanism – resource) can facilitate more intensive and sensitive conversations (outcome). This might be challenging for the befriender in terms of their expertise and capabilities, and therefore hinder their ability to deliver an effective service (mechanism – reasoning), subsequently threatening the longevity of the relationship (outcome). |
| **PT 3:** If the befriending service trains befrienders, particularly those with no previous relevant experience (context), then befrienders are likely to feel better prepared and more equipped in their role (mechanism – reasoning), as they will gain good knowledge and insight into their role as a befriender and into the illness of the user-group of the service (mechanism – resource) so therefore will become more competent and confident in delivering an effective service (outcome). Moreover, befrienders’ ability to dissociate the role from their personal life will be strengthened which is likely to increase the retention rate and enhance the longevity of their relationship with service users (outcome). Training also allows for befrienders to develop an awareness of resources available to them and establish a support network with other befrienders and the managerial staff in the organisation (mechanism – resource). Additionally, where a befriender has prior relevant experience of the health condition of service users (context), training can provide more insight into the illness and opportunities to enhance pre-existing skill-set and establish new ones that can aid in effective service delivery (outcome). |
| **PT 4:** If a volunteer befriender (as opposed to a befriender that receives monetary compensation) is provided to service users, particularly those with a physical, sensory or cognitive impairment (context), then service users recognise that their befriender is choosing to spend time with them rather than being under any professional obligation or for financial gain (mechanism – reasoning). This facilitates an environment where an emotional connection can be easily developed as the engagement between both parties would feel more natural and friendship-like (outcome). Service users are likely to perceive their befriender as more genuine (mechanism – reasoning) and a display of this promotes reciprocity (outcome) as befrienders would feel appreciated and satisfied in their role and therefore motivated to continue delivering the service (mechanism – reasoning). However, in circumstances where service users do not feel satisfied with the befriending relationship (context), knowledge of the voluntary nature of their befriender might hinder them from taking any action to resolve this as they would feel guilty (mechanism – reasoning). This could hinder the development of a meaningful relationship and exacerbate feelings of loneliness in service users (outcome). |
| **PT 5:** If a befriender is delivering the service with monetary compensation, then they are more likely to be professional in their approach to service delivery (outcome). Service users would have more autonomy over how the service is delivered to them (mechanism – resource) and can adapt the service to suit their pace, demands and expectations (outcome). Moreover, the relationship with their befriender is likely to be long-term as the payment for befrienders provides more security and assurance of the befriending visits (mechanism – resource). In circumstances where service users are vulnerable (context), utilising a paid befriender is more reassuring that the appropriate care would be provided to service users due to the perception that paid befrienders are more likely to adhere to the rules and regulations of the service (mechanism – reasoning). Additionally, the monetary compensation is unlikely to negatively affect how a befriender is perceived by service users or hinder the development of a meaningful relationship in circumstances where befrienders have a caring disposition (mechanism – resource) and are perceived to be genuine by service users. However, payment for befrienders is likely to breed higher expectations and demands from service users (mechanism – reasoning), particularly those with reduced mobility and having physical needs as a result of a limiting health condition, e.g. visual impairment (context). Unfulfillment of these expectations could lead to service users feeling dissatisfied and potentially withdrawing from the befriending relationship (outcome). |
| **PT 6:** If the befriending service is delivered long-term, especially to service users who have a sensory, cognitive and/or physical impairment that limits their mobility and causes them to be refined to their home (context), this allows for familiarity to develop as both parties would gain better knowledge of each other which subsequently increases the capacity of befrienders to deliver a person-centred service to suit the needs of service users (outcome). Moreover, continuity in the befriending relationship strengthens the connection between both parties and facilitates the development of trust and reciprocity. Hence, it is likely that the relationship will deepen beyond the superficial level and into a type of friendship which will help alleviate feelings of loneliness in service users (outcome). Additionally, the befriending visits provide service users something to look forward to and reassures them that there will be someone checking up on them (mechanisms – reasoning) thus enhancing their emotional and psychological wellbeing (outcome). |
| **PT 7:** If the befriending service is delivered on a one-to-one basis, particularly to individuals who have a sensory, cognitive or physical impairment (context), then the likelihood of a meaningful relationship being established is enhanced because the delivery format provides reduced stimulation and privacy (mechanism – resource) which can stimulate focused-conversations and encourage the development of trust (outcome). Subsequently, there is likely to be sharing of personal information which will strengthen the bond between both parties and service users will feel more listened to and valued by their befriender (mechanism – reasoning). Moreover, befrienders would gain more knowledge of service users which would equip them to be able to deliver a service that is more attuned to their needs (outcome). |
| **PT 8:** If a service user, particularly those with a sensory or cognitive impairment (e.g. dementia) (context), is visited by the same befriender (as opposed to different befrienders), then it is likely that reciprocity would be established as continuity in the relationship allows both parties to gain a deeper knowledge of each other which enhances the quality of the relationship and strengthens its depth (outcome). Moreover, in circumstances where service users have communication difficulties (context), befrienders would develop better knowledge and understanding of the idiosyncratic communication style of service users which enhances the communication and likelihood of the befriender meeting their needs (outcome). Additionally, the health impairments in service users increases their vulnerability, hence the importance in the same befriender visiting as this provides stability, routine and security (mechanism – resource) which subsequently facilitates the development of trust and stimulates the development from a professional relationship to a more genuine friendship (outcome). |
